# Point-of-Care Ultrasound Unveiling Rotator Cuff Injuries in the Emergency Department: A Case Series

**DOI:** 10.7759/cureus.47665

**Published:** 2023-10-25

**Authors:** Lauren Selame, Lindsay Walsh, Madeline Schwid, Nour Al Jalbout, Morgan R Gray, Munaa Dashti, Hamid Shokoohi

**Affiliations:** 1 Emergency Medicine, Brigham and Women's Hospital, Harvard Medical School, Boston, USA; 2 Emergency Medicine, Massachusetts General Hospital, Harvard Medical School, Boston, USA; 3 Emergency Medicine, Brigham and Women’s Hospital, Harvard Medical School, Boston, USA; 4 Emergency Medicine, Amiri Hospital, Kuwait City, KWT

**Keywords:** emergency department utilization, arthrocentesis, shoulder joint pain, rotator cuff tears, point-of-care-ultrasound

## Abstract

Acute shoulder pain is a common ED presentation with a wide range of pathologies that are often initially investigated with radiography. However, diagnosing rotator cuff injuries often requires further imaging for proper diagnosis and management. Bedside shoulder ultrasound is an application that allows for the evaluation of ligaments and tendons in addition to bony structures, all while utilizing direct patient feedback of focally tender areas, expediting diagnosis and therapeutic intervention. In this case series, we discuss our evaluation of patients with suspected rotator cuff pathology and the practice of using the stepwise shoulder ultrasound protocol. Four cases are presented that illustrate the use of shoulder ultrasound in diagnosing biceps tendon injury, supraspinatus tear, chronic supraspinatus tear with hemarthrosis, and subacromial-subdeltoid bursitis. This narrative highlights the valuable role of shoulder ultrasound for the expedited diagnosis and management of patients whose initial shoulder radiographs do not indicate any bony abnormalities.

## Introduction

Acute shoulder pain is one of the most frequent upper extremity complaints among patients presenting to the ED [1]. Shoulder pain is estimated to encompass 14.7 cases per 1,000 patients annually [2]. Shoulder pathologies, such as adhesive capsulitis and rotator cuff injuries, are diagnosed first by history and physical examination and are then confirmed by various imaging modalities. While some diagnoses can be confirmed in the ED with plain radiography, injuries to the rotator cuff apparatus are typically diagnosed by physical examination with plain radiographs used to rule out fractures or dislocations. The American College of Radiology recommends shoulder radiography as the initial imaging modality to evaluate atraumatic shoulder pain. However, studies suggest that shoulder radiography in the ED may be overutilized and of limited diagnostic benefit, except when ruling out fractures or dislocations [3]. This often leads to patients being discharged with recommendations for supportive care and follow-up for potential outpatient MRI, the gold standard for diagnosing rotator cuff injury, arthrography, or ultrasound for a definitive diagnosis [4,5].

Ultrasound is increasingly accessible in EDs, orthopedic clinics, and even resource-limited clinical settings. Consequently, ultrasound may provide a means for faster definitive diagnosis and proper outpatient follow-up [6]. For patients presenting to the ED with shoulder complaints, the use of musculoskeletal (MSK) ultrasound facilitates direct visualization of focally tender areas and may increase patient satisfaction [7]. Furthermore, the ultrasound scan may allow ED clinicians to diagnose a wide range of traumatic and atraumatic shoulder pathologies without the use of radiation or more invasive, costly, or time-sensitive imaging modalities [2-8].

Recent studies have shown that emergency medicine resident physicians with no prior training in shoulder ultrasound can successfully diagnose a number of non-fracture shoulder pathologies using point-of-care ultrasound (POCUS) after a brief educational intervention [9]. Shoulder ultrasound is a practical and accessible imaging modality that can expedite the diagnosis of rotator cuff tendon pathology in the ED, particularly for patients whose shoulder radiographs do not show bony abnormalities such as fractures, joint separations, or dislocations [10].

In this case series, we discuss our practice of using shoulder POCUS in the ED to expedite the diagnosis of rotator cuff injuries. In this report, we emphasize the valuable role of shoulder POCUS in patients who present to the ED with acute shoulder pain and initial shoulder radiographs without bony abnormalities.

Shoulder ultrasound technique

Shoulder POCUS was performed according to the stepwise shoulder ultrasound protocol [11]. This protocol introduces the acromioclavicular (AC) joint, biceps tendon, subscapularis, impingement, and supraspinatus (ABSIS) ultrasound protocol, which provides a stepwise approach to performing and interpreting shoulder ultrasound. The ABSIS protocol guides clinicians through a systematic ultrasound evaluation of the shoulder tailored to patient complaints. It emphasizes the evaluation of rotator cuff injuries, such as tendon tears, ruptures, or tendinopathies, as well as bony anatomy. All ultrasound examinations were performed with the probe-oriented cephalad or toward the patient's right. If shoulder dislocation is not on the clinical differential, the protocol begins with the linear probe and examines the AC joint and clavicle, biceps tendon, subscapularis tendon, supraspinatus tendon and subacromial space, humeral head, and glenohumeral joint from the posterior aspect (typically performed using the curvilinear probe) [11]. If shoulder dislocation is on the differential, it is recommended to start initially with the view of the glenohumeral joint; however, these cases are not presented in this case series.

## Case presentation

Case 1: long head of the biceps tendon (LHBT) injury

**Figure 1:**
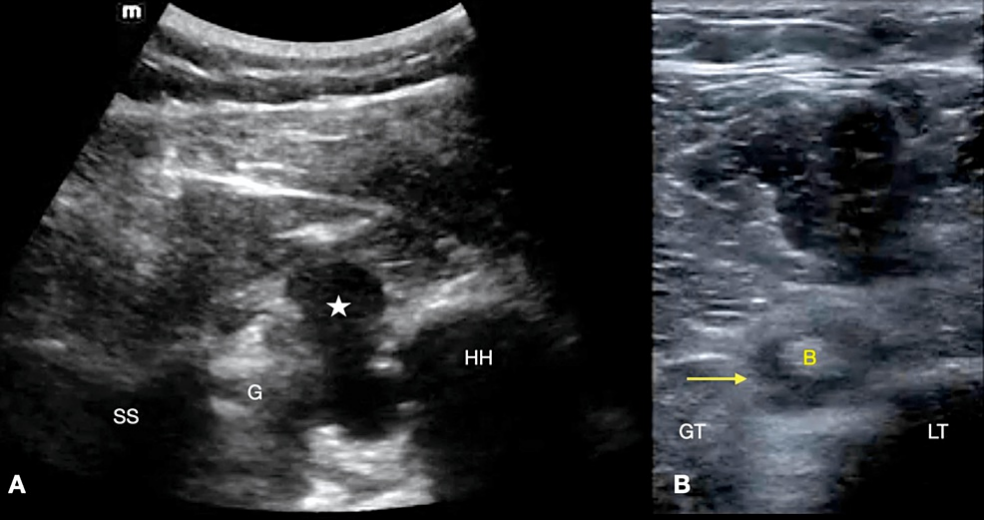
(A) Transverse view of the posterior glenohumeral joint using a curvilinear probe, demonstrating an effusion. (B) Transverse view of the biceps tendon using the linear probe showing fluid around the tendon (A) star: effusion, SS: scapular spine, G: glenoid, HH: humeral head (B) arrow: fluid, B: biceps tendon, LT: lesser tuberosity, GT: greater tuberosity

A 43-year-old right-handed male with a history of hypertension presented with traumatic, severe left shoulder pain after lifting multiple 130-pound objects at work. Examination of his left shoulder showed focal tenderness over the lateral left acromion and LHBT with limited active flexion and abduction of the left shoulder to 40 degrees. He had positive Neer's and Hawkin's tests. Radiographs of the left shoulder were negative for fracture or dislocation. Dedicated POCUS demonstrated anechoic fluid surrounding the LHBT and an intra-articular effusion (Figure 1). The patient was discharged from the ED with a plan for pain control and outpatient orthopedics follow-up. He underwent an outpatient MRI that demonstrated LHBT and supraspinatus tendon injury. He underwent surgical repair four months after his ED visit and recovered without complications.

Case 2: acute supraspinatus tear

**Figure 2:**
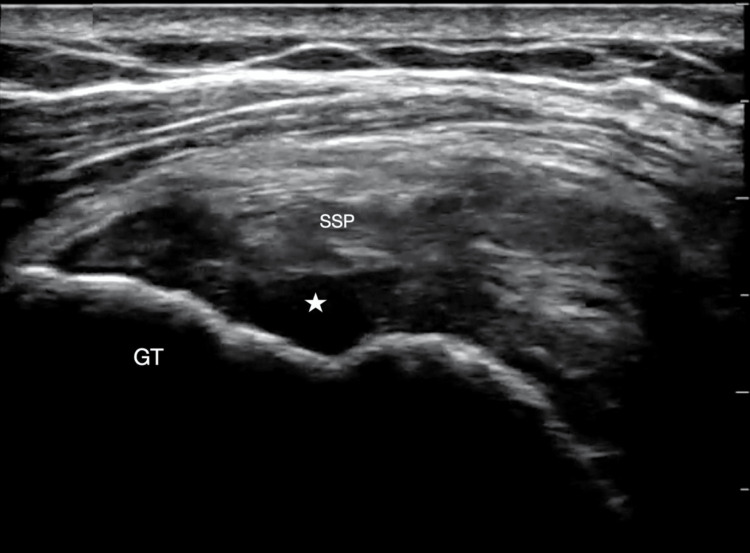
Transverse view of the supraspinatus tendon using a linear probe showing disruption of the supraspinatus tendon and surrounding fluid Star: fluid, SSP: supraspinatus tendon, GT: greater tuberosity

A 79-year-old right-handed male with a history of diabetes presented to the ED with acute onset right shoulder pain that started one day prior. He endorsed severe pain in the right shoulder that worsened with abduction and improved with rest in a neutral position. His exam was notable for focal tenderness over the right shoulder joint and pain with abduction past 50 degrees. He was otherwise neurovascularly intact. A right shoulder radiograph was negative for acute fracture or joint dislocation. POCUS of the right shoulder was performed, demonstrating signs of a supraspinatus tear with surrounding anechoic fluid and tendon discontinuity (Figure 2). He was referred to outpatient sports medicine and later underwent a right shoulder MRI that showed a focal, full-thickness tear of the anterior supraspinatus leading edge fibers as well as partial thickness tearing of the infraspinatus and subscapularis tendons. He improved with symptomatic treatment and non-operative management.

Case 3: chronic supraspinatus tear and glenohumeral joint hemarthrosis

A 96-year-old female presented to the ED for severe pain in her right shoulder that started six months after a mechanical fall. Her pain typically improved with tramadol, glenohumeral joint corticosteroid injections, and glenohumeral joint aspiration. At the time of presentation, her pain was more intense and worsened with movement. She noted multiple days of increased use of the affected extremity just prior to presentation. She denied numbness, paresthesias, fever, chills, or recent trauma. She was otherwise in good health and remained active. On examination, a palpable effusion on the right anterior shoulder was appreciated without evidence of erythema, warmth, or deformity. Her range of motion was limited due to pain, particularly with abduction and external rotation of the right shoulder.

**Figure 3:**
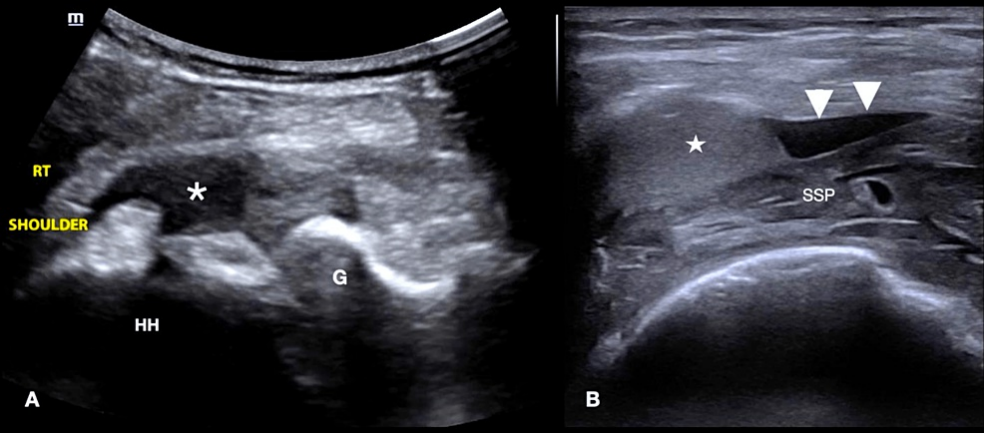
(A) Transverse view of the posterior glenohumeral joint using the curvilinear probe demonstrating mixed echogenic effusion. (B) Transverse view of the supraspinatus tendon using the linear probe showing surrounding fluid and hematoma (A) star: effusion, HH: humeral head, G: glenoid (B) star: hematoma, arrowheads: fluid, SSP: supraspinatus tendon

One month prior, she presented with a similar history of worsening pain. A radiograph at the time showed no fracture or dislocation. On the current presentation, a POCUS was performed, which revealed a supraspinatus tendon injury with a surrounding heterogeneous effusion consistent with a hematoma and glenohumeral joint hemarthrosis (Figure 3). A POCUS-guided arthrocentesis of the right shoulder was performed, in which 25 mL of blood was removed from the joint space. The patient noted significant improvement in pain and range of motion shortly after the procedure. She was subsequently discharged home with outpatient orthopedics follow-up.

Case 4: subacromial subdeltoid bursitis

**Figure 4:**
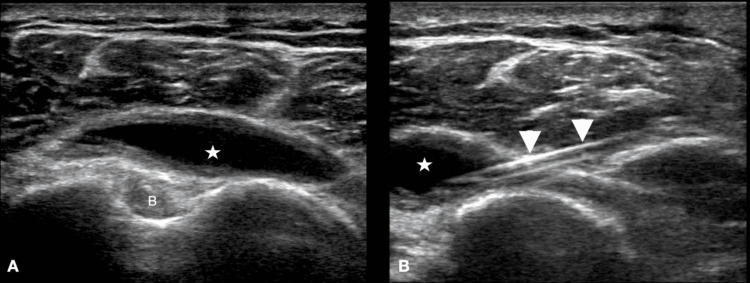
(A) Transverse view of the bursa sac using the linear probe, overlying the LHBT when the patient is in adduction and supination. (B) Needle directed into the bursa (A) star: fluid within the subacromial-subdeltoid bursa, B: LHBT (B) arrowheads: needle, star: fluid within the subacromial-subdeltoid bursa

A right-hand-dominant male in his 60s presented with four months of atraumatic left shoulder pain. He had previously been evaluated for this pain in the ED but noted acute worsening prior to presentation after lifting a heavy object. He had no history of intra-articular injections. The pain was severe and worse with movement in all directions. He denied swelling, erythema, numbness, paresthesias, weakness, or fevers. The examination was notable for tenderness over the LHBT insertion site with palpable crepitus of the left shoulder. He had a full range of motion in the left shoulder, but pain was noted with any active or passive motion. He was neurovascularly intact without evidence of swelling or erythema. Based on the presentation and clinical exam, there was a low concern for a septic joint. A previous radiograph of the shoulder showed degenerative changes in the AC joint. A shoulder POCUS was performed, which demonstrated subacromial-subdeltoid bursitis (Figure 4). The patient received a steroid injection of triamcinolone 40 mg/mL into the bursa and was discharged home with outpatient follow-up in the sports medicine clinic.

## Discussion

Case 1: LHBT injury

LHBT injuries commonly cause anterior shoulder pain and usually result from direct or indirect trauma [12]. The LHBT originates at the supraglenoid tubercle of the scapula and superior glenoid labrum. The tendon contributes to the stability of the shoulder as it courses along the glenohumeral joint, eventually joining with the short head of the biceps tendon [13,14]. LHBT injuries are usually secondary to rotator cuff disease rather than traumatic injury. This is in contrast to a distal biceps tendon rupture, which typically results from excessive eccentric force [15].

Shoulder ultrasound can readily identify the LHBT in the intertubercular groove (bicipital sulcus) beneath the transverse humeral ligament. The tendon is then traced upward to its origin at the supraglenoid tubercle. In a study by Skendzel et al., ultrasound was found to be 88% sensitive and 98% specific for diagnosing a full-thickness tear. The major disadvantages of using ultrasound in these cases include low detection of partial tears and the inability to visualize intra-articular lesions. If fluid is identified in the biceps sheath, it suggests biceps tendonitis or tear rather than rotator cuff injury. Intra-articular fluid can be present with biceps tendon injuries, as the biceps tendon sheath is derived from the extension of the glenohumeral joint capsule [16-19].

Case 2: acute supraspinatus tear

The supraspinatus tendon is one of the rotator cuff tendons that originates from the supraspinous fossa at the superior portion of the spine of the scapula and inserts on the greater tuberosity of the humerus. In patients older than 60 years, a non-traumatic rotator cuff tear may be regarded as a consequence of tendon degeneration, with a prevalence of up to 30% in this population. Since the supraspinatus and infraspinatus tendon insertions overlap, large tears of the supraspinatus tendon tend to extend to the infraspinatus tendon, similar to our case. Conservative management with a non-operative approach has similar long-term outcomes to operative management [20-23].

Shoulder ultrasound can readily identify the supraspinatus tendon, which laterally has a beak-shaped configuration that attaches to the greater tuberosity. Supraspinatus tendon tears can be partial or full thickness, with the anterior border being the most common site of tears. A hypoechoic or anechoic defect with tendon retraction confirms the diagnosis of a full-thickness tear. Additional findings include the presence of fluid in the subacromial/subdeltoid bursa or the glenohumeral joint [24,25]. Ultrasound is highly sensitive (88%) and specific (93%) in the diagnosis of supraspinatus tears but is less accurate in the differentiation of partial-thickness versus full-thickness tears [26,27].

Case 3: chronic supraspinatus tear and glenohumeral joint hemarthrosis

Spontaneous hemarthrosis has been reported previously with rotator cuff tears. In some instances, ultrasound can distinguish hemarthrosis from non-bloody shoulder effusions based on echogenicity and the presence of displaceable echogenic reflectors. The presence of increased flow using the power Doppler or color Doppler functions on most ultrasound machines can also help differentiate these diagnoses. Compared to MRI, ultrasound is more sensitive to low volume and blood concentration and can be used to detect active bleeding [27-29]. It is also important to note that a glenohumeral joint effusion may produce fluid within the biceps tendon sheath, as the tendon sheath communicates with the aforementioned joint space.

The initial treatment of hemarthrosis includes conservative management with immobilization, ice, compression, and pain control. Arthrocentesis can aid in diagnosis and provide symptomatic relief by reducing pressure caused by the effusion [30]. When performing ultrasound-guided shoulder arthrocentesis, the posterior exam is a proper approach [31]. Specifically, the posterior examination involves palpating the scapular spine at the level of the humeral head and placing the linear probe in the transverse plane to visualize the infraspinatus tendon in the long axis. The examination also allows visualization of the spinoglenoid notch and posterior labrum. As demonstrated in this case, ultrasound can also be used for procedural guidance when performing a shoulder arthrocentesis and has been found to increase success rates [31,32].

Case 4: subacromial subdeltoid bursitis

Subacromial-subdeltoid bursitis is a common etiology of shoulder pain that results from inflammation of the bursa under the acromion process, often due to overuse and repetitive movements. On ultrasound, the bursa is seen as an anechoic fluid-filled structure with a hyperechoic wall. Ultrasound can detect thickening of the bursa, as seen in bursitis, and can be used to guide therapeutic injections [33].

MSK ultrasound in the ED has shown promising results in diagnosing and managing articular and periarticular injuries, such as tendon and ligamentous injuries [6,8,9,34-36]. In this case series, we discuss the use and findings of shoulder ultrasound as an inexpensive, accessible, and practical imaging modality for patients with acute shoulder pain suspected of rotator cuff injuries.

Although patients often seek radiography for diagnosis and guidance of treatment for shoulder pain, completion of radiographs does not lead to significant patient benefit. For patients with shoulder pain, using ultrasound expedites diagnosis, streamlines management plans, and allows for safer procedural interventions. Using ultrasound to identify an anatomical site where bursitis, glenohumeral joint effusion, or hemarthrosis is present can greatly improve practitioner comfort, competence, and success in performing shoulder aspiration, as well as steroid injections, if clinically necessary. Considering the increasing time and financial constraints placed on many EDs throughout the country, proficiency in shoulder ultrasound may lead to reduced length of stay and better targeting of patients who require outpatient follow-up [2]. Furthermore, diagnosis of rotator cuff injury and/or performance of steroid injections or aspirations may reduce the need for and number of outpatient follow-up appointments necessary [37,38].

Unlike a knee ultrasound in which emergency physicians have become adept given the frequency of knee arthrocentesis, the shoulder ultrasound remains a less common practice for shoulder pathologies and procedures [38]. Although landmark-based approaches are taught for shoulder arthrocentesis, the use of shoulder ultrasound to identify and guide arthrocentesis or discover an alternative etiology of pain is a field of ultrasound that has not yet been tapped. Studies have shown higher needle placement accuracy is achieved with a posterior approach to the glenohumeral joint when preparing for arthrocentesis, compared to the anterior approach. Regardless, both approaches appear well-tolerated, and complications such as hematoma, infection, pneumothorax, or injection into the tendon are uncommon [39,40].

## Conclusions

In the presented cases, we demonstrated the unique role of the shoulder ultrasound as both a diagnostic and procedural modality for a number of different causes of shoulder pain, as seen and evaluated in the ED. For these patients, we were able to scan specific anatomic areas of pain and discomfort, leading to prompt, visual diagnoses at the bedside. Our patients were relieved to have specific and readily available information regarding the cause of their pain. In our experience, POCUS use in evaluating shoulder pain is instrumental. It can potentially improve bedside care, decrease ED length of stay, and reduce the need for costly and time-consuming imaging modalities. Further research is needed to optimize the use of shoulder ultrasound for different pathologies in ED practice.
